# Geographical variations in the correlates of blood donor turnout rates: An investigation of Canadian metropolitan areas

**DOI:** 10.1186/1476-072X-8-56

**Published:** 2009-10-13

**Authors:** PJ Saberton, Antonio Paez, K Bruce Newbold, Nancy M Heddle

**Affiliations:** 1 School of Geography and Earth Sciences, McMaster University, Hamilton Ontario, Canada; 2 Clinical Epidemiology and Biostatistics, McMaster University, Hamilton Ontario, Canada

## Abstract

**Background:**

Like other countries, Canada's population is aging, and the implications of this demographic change need to be better understood from the perspective of blood supply. Analysis of donor data will help to identify systematic patterns of donation and its correlates.

**Data:**

Geo-coded blood donor and donor clinic data are provided by Canadian Blood Services. Blood donor data is provided for the fiscal year 2006-2007 indicating the total number of donors for each Canadian postal code, excluding the province of Québec. Potential correlates of blood donation are selected based on social and economic characteristics, as well as descriptors of city size and geographical location in the urban hierarchy measures of accessibility, and capacity of donor clinics.

**Methods:**

Data is aggregated to *n *= 3,746 census tracts in 40 Census Metropolitan Areas (CMA) across the country. The number of donors per population in a census tract is regressed against the set of potential donation correlates. Autocorrelation is tested for and results adjusted to provide parsimonious models.

**Results:**

A number of factors are found to influence donation across the country, including the proportion of younger residents, English ability, proportion of people with immigrant status, higher education, and a population-based measure of accessibility.

**Conclusion:**

While a number of correlates of blood donation are observed across Canada, important contextual effects across metropolitan areas are highlighted. The paper concludes by looking at policy options that are aimed toward further understanding donor behaviour.

## Background

Blood products play an important role in modern medical procedures that can both save and extend life. It is therefore critical for any health system to ensure that the volume of blood supply is sufficient to satisfy the demand, and Canada is no exception. In Canada, with the exception of Québec, the organization responsible for collecting and distributing the national blood supply is Canadian Blood Services (CBS). This is a national, not-for-profit charitable organization that operates 40 permanent collection sites and more than 20,000 donor clinics annually, and is charged with overseeing the safety of the blood supply, educating the public and recruiting blood donors. CBS operates under guidelines that establish that blood donation is a voluntary endeavour open to anyone in good health, 17 years or older, and weighing at least 50 kg [1]. To ensure the safety of the blood supply, a number of restrictions are implemented by CBS that essentially define the meaning of being in "good health". Thus, there are a number of disqualifying conditions that impede a potential donor from becoming one, including having tested positive for West Nile Virus (WNV) and infectious diseases [2]. Despite these safety restrictions, there is still a large pool of potential donors to support the system, estimated at about "12.5 million eligible donors in Canada" [3]. The reality, however, is that donor participation tends to be limited due to concerns for health effects and a general lack of education on the part of the public regarding the importance and lack of risk involved in donating blood. There is evidence, for instance, that up to twenty-five percent of Canadians mistakenly believe that donating blood is less than completely safe [4]. The negative effect becomes evident when considering the disappointingly low number of actual donors: of the potential pool, it is estimated that "only 3 per cent of adult Canadians donate blood while virtually all Canadians will need blood or blood products in their lifetime" [5]. While the system has so far proved sufficient (i.e. medical procedures are not routinely cancelled because of blood being unavailable), relying on such a small percentage of the Canadian population to provide the amounts of blood required to sustain the system in the long term is not advisable (A. Steed, personal communication, July 7, 2008). The limitations of placing the burden of sustaining the blood supply on a small number of donors becomes particularly salient within the context of an aging society, such as Canada's, for which projections indicate that one in five people will be at least 65 years old by 2021, and approximately 6% of the population will be older than 80 [6]. Given general population health trends, this strongly suggests that the actual pool of donors will shrink in the future, at the same time that the number of potential users of blood products will almost certainly grow.

To effectively achieve a reliable supply of blood, an initial large enrolment of first time donors and their subsequent retention as repeat donors is needed. Increasing the total number of Canadians donating blood for the first time is necessary to help meet national demands, while retaining these donors is important because of the higher cost of continuously recruiting new donors. Also, repeat donors provide a safer supply of blood, with a lower incidence of infectious diseases [7]. The key to attracting new donors (and subsequently repeat donors) requires a number of actions, which include: 1) Developing economically efficient and effective marketing campaigns; 2) Finding ways of increasing the convenience of donating, by optimizing operation hours, locations, etc.; and 3) Educating the public about the importance, ease, and lack of risks of giving blood. These are all required elements that could encourage Canadians to make blood donation part of their lifestyles.

**Figure 1:**
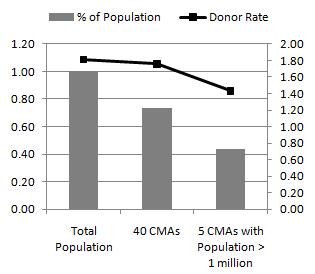
**Donor rates by geographical context**.

The challenge of achieving these objectives is underlined by the difficulties of reaching and serving the large, heterogeneous, target market of all eligible Canadian donors. In order to be efficient and effective, recruitment plans aimed at increasing donor levels must to be targeted at untapped populations, or those that are most likely to donate in each part of the country. In this regards, it is known that the patterns of donation vary between cities and regions. Figure 1 shows, for example, the proportion of the Canadian population (excluding Québec) living in Census Metropolitan Areas, or CMAs, defined by Statistics Canada as one or more contiguous municipalities, totalling at least 100,000 inhabitants, and situated around a major urban core with a population of at least 50,000. As seen in the figure, over 70% of the population live in CMAs and slightly above 40% in the 5 largest (population greater than 1 million) metropolitan areas. And, while the overall donor rate for the 40 CMAs is not very different from the population rate (calculated as number of donors over total population, not only eligible population), it can be seen that the largest metropolitan areas in the country tend to have substantially lower donor rates. There are a number of reasons why variations in donation patterns could arise, including varying economic, social, cultural, demographic, and historical factors of a given region, which may affect the motivating forces towards whether or not to donate [8-10]. More generally, there is a noticeable amount of heterogeneity with respect to patterns of volunteering inter-provincially, as well as between varying sized cities [11,12]. This suggests that residents in different areas may display, in addition to varying demographic attributes, different incentives and outlooks on volunteering and health. Therefore, there is a need to better understand the correlates of blood donation. This is a question of considerable interest since the answer may help to identify planning objectives, as well as to define whether outreach and service plans are developed nationally, regionally, or even locally.

With the above considerations in mind, the objective of this paper is to investigate a wide array of correlates of donor turnout rates at the census tract level in order to determine the factors that influence the number of donors (a census tract is defined by Statistics Canada as a small, relatively stable area, usually with a population between 2,500 and 8,000 inhabitants). Furthermore, we also seek evidence of geographical segmentation of the Canadian population in terms of blood donation behaviour. More concretely, our objective is to determine whether the socio-demographic profile of various cities display patterns of similarity based on city size and region of the country, or whether on the contrary, there are geographical variations in the way donation is influenced by various factors. Besides the work of van der Pol et al. [13], there is scarcely any international research into this topic. In Canada, a recently released report explores the situation in Québec [14], but the present paper is the first effort to investigate the situation in the rest of the country. Statistical analysis of donor data helps to identify systematic patterns of donation and its correlates, which in turn provides insights into the potential of various incentives, levels of service, or marketing practices, including whether these need to be directed to a particular city based on its demographic profile, geographic location, or size.

## Data

**Figure 2:**
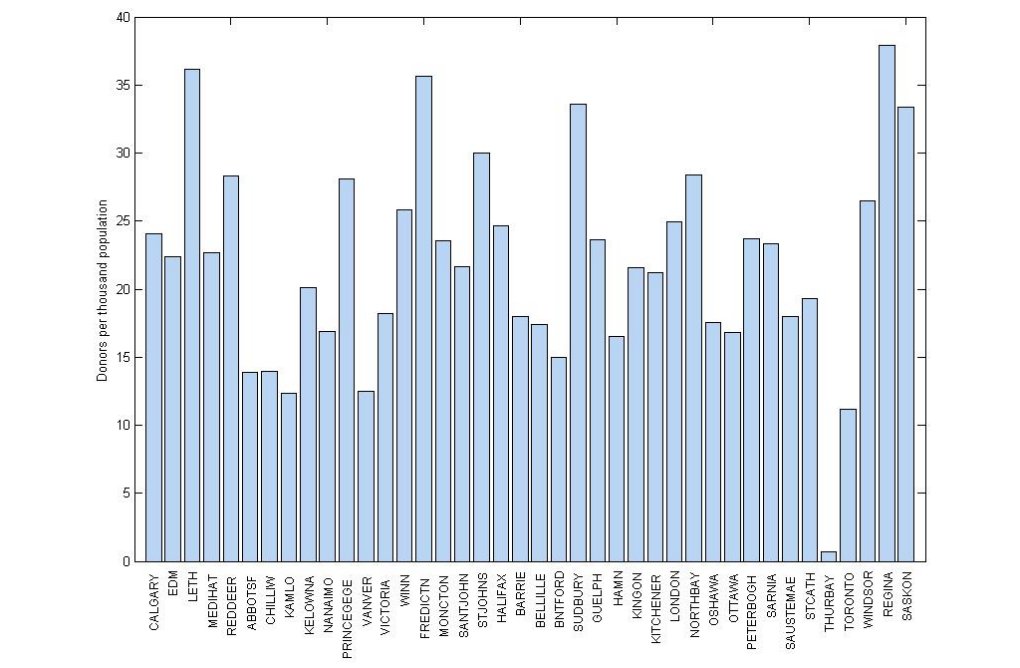
**Donors per thousand population in target CMAs**.

The research reported in this paper is based on geo-coded donor and donor clinic data provided by Canadian Blood Services for the purpose of this study. Blood donor data were provided for the fiscal year 2006-2007 in aggregate form, indicating the total number of donors for each Canadian postal code, by place of residence. Canadian postal codes range in size from approximately zero to more than 60,000 homes, averaging about 8,000 households for each postal code. Converting this data to the number of donors for each Canadian census tract allows all donor and donor clinic information to be linked to 2006 Census attribute information. The population consists of *n *= 3,746 census tracts in 40 Census Metropolitan Areas across the country containing a total of 310,767 unique donors. Number of donors per thousand population are shown in Figure 2 for each of the 40 CMAs in the analysis further illustrating the important differences between donor turnout rates in each location. Donor clinic data were provided in point-based form with longitude and latitude references. The clinic database is exhaustive, and includes a total of 19,671 clinics at approximately 1,600 unique locations.

Potential correlates of blood donation are selected based on social and economic characteristics that have been demonstrated by previous research to correspond to high or low donor turnout. Other variables describe the size of metropolitan areas or are introduced to account for fixed (city-specific) effects in the form of dummy variables. The objective of these dummy variables is to capture any contextual variation specific to a metropolitan area that is not attributable to other explicative factors. Finally, we also introduce measures of accessibility and capacity of donor clinics (see Table 1).

**Table 1:** Variables and Definitions

**Variable**	**Definition and units**	**Min**	**Max**	**Mean**	**SD**
Dependent variable					

DONORS	Donor rate in census tract	1	683	82.96	60.38

Socio-economic and demographic characteristics					

POP15TO24	Proportion of population of 15-24 years of age in census tract	0.04	0.66	0.14	0.03
POP25TO54	Proportion of population 25-54 years of age in census tract	0.15	0.77	0.44	0.06
POP55TO64	Proportion of population 55-64 years of age in census tract	0.02	0.26	0.11	0.03
POP65+	Proportion of population of 65+ years of age in census tract	0.00	0.68	0.13	0.07
ENGLISH	Proportion of English-speaking population in census tract	0.03	1.00	0.69	0.20
IMMIG	Proportion of immigrant population in census tract	0.00	0.82	0.26	0.18
UNEMP	Proportion of unemployed population in census tract	0.00	0.12	0.03	0.01
HEALTHOCC	Proportion of population in census tract in health-related occupations	0.00	0.13	0.03	0.01
HIGHED	Proportion of population in census tract with bachelors degree or higher	0.00	0.68	0.21	0.11
Ave Household Income	Average household income ($10,000)	1.73	81.08	7.91	3.96

Geographic variables					

CMAPOP	Total CMA population (1,000,000s)	0.06	5.11	1.93	1.99
City Name	City-specific dummy variable				

Service variables					

Pop Based Access	Proportion of population-based accessibility within 4 km band (beds-hour/1000 people)	0.00	481.74	34.63	61.53

Socio-economic and demographic variables (age, income, employment, and education) are obtained from the Census on the basis of census tract aggregations. We collect variables describing the demographic profile of census tracts. Hollingsworth and Wildman [8] and Burnett [9] indicate that donation rates vary in relation to the number of individuals present for different age groups. In order to capture variations due to demographic structure, four age groups were defined as follows. First, the population of individuals between the ages of 15 and 24 is defined as the 'school aged' population, to include people who are exposed to CBS' school learning programs and on-site donor clinics. While the minimum age for donation is 17, Statistics Canada reports population counts in groups, with the closest matching groups beginning at age 15. The population of individuals aged 25 to 54 is characterized as 'working age donors', who may have less time to donate, and therefore would have lower donor turnout rates. The pre-retirement cohort includes those aged 55 to 64, are considered senior donors. The fourth age group is comprised of the population of individuals who are 65 and over. These donors are more likely to receive health-related deferments and are potentially large users of the blood supply. A large population of this demographic is expected to have a negative effect on the donor rate.

A variable regarding language is also included. This variable is meant to test whether individuals whose first official spoken language is English are more responsive to CBS educational programs. Thus, the total number of English-speaking people for each census tract is selected as an explanatory variable. Given the importance of immigration to the makeup of the Canadian population, the immigrant population is considered a relevant variable as well. The potential effect of this variable is ambiguous. Lacking other evidence, there are plausible explanations both in favour and against donor turnout, for example, if immigrants are more civically minded towards their host country, or on the contrary come from places where volunteering is less well regarded or blood donation is considered more risky.

Caro and Bass [15] found that adults who are employed are more likely to volunteer their time than those who are unemployed. Thus, it is expected that the variable for the total number of unemployed individuals will correlate negatively with donor turnout. Individuals employed in health related fields are exposed to the importance of blood donation in life-saving and life-prolonging procedures, either through training and education, or from work-related experiences. Thus, the number of people employed in health related professions in each CT is expected to have a positive impact on donor turnout. Since rates of donation are known to increase with level of education [16], the number of individuals with a college certificate or higher is included as an explanatory variable. The idea of wealthier individuals being more likely to donate also follows most models including Jirovec and Hyduk [17], and income is therefore expected to have a positive correlation with donation patterns.

In order to identify potential variations in blood donation patterns, we introduce the total population of each Census Metropolitan Area as a macro-level descriptor of each area. This variable is used to test the proposition that there are systematic variations in the correlates of donation following city size.

Finally, we also introduce variables that describe the levels of service provided by blood donor clinics around the country, and how accessible the services are. Accessibility variables are calculated using the two-step floating catchment area proposed by Radke and Mu [18] and applied by Wang and Luo to identify health professional shortage areas [19]. Since some donor clinic locations are permanent and others are temporary or ambulatory, and their levels of services vary widely, instead of simply recording presence we consider for each donor clinic location the number of beds available at the event (*B*_*i*_) and the total service time in hours (*T*_*i*_). With this, a measure of service is obtained as total number of beds-hours available at the location. The two-step catchment area procedure begins by centering a catchment area on donor clinic location *i *= 1, 2, ..., *n*_*i *_(the number of clinics), and searching all census tract centroids within a threshold (Euclidean) distance *d*_0 _of that location. The level of service at the location is calculated as follows:

(1)

where *P*_*j *_is the population in the census tract *j *in thousands. The level of service is thus measured in beds-hours per thousand people. The second step of the procedure then consists of "floating" the catchment areas to census tract centroid *j *= 1, 2, ..., *n*_*j *_(the number of census tracts) and calculating the level of service accessible to each census tract by adding the level of service of all donor clinics within a distance *d*_0 _of census tract *j*:

(2)

Accessibility is calculated based on residential population (ACCPOP). With regards to the selection of a critical distance, it has been noted that the use of travel behaviour information can yield valuable information to determine the catchment areas [20]. Unfortunately, very little is known about the travel behaviour of potential donors and no data are available to assess it. In particular, donors are coded by place of residence, but it is unknown whether they travelled from their home, workplace, or other location at the time of donation. Lacking other information, we decide to calculate the accessibility indicators using bands between 1 km and 10 km in 1 km increments. Band selection is based on the statistical fit and properties of the models, as described below.

## Methods

To implement the analysis, the number of individual blood donors divided by the census tract population is taken as the dependent variable to estimate the coefficients of a log-linear regression model. The specification used in this analysis is the following regression model (in matrix form):

(3)

where ***D ***is the vector of donor rates for each census tract; **X **is the matrix of explanatory variables; ***β ***is the vector of regression coefficients; and ***ε ***is a vector of independent and identically distributed error terms. Given the geo-coded nature of the data, we also test for potential autocorrelation in the residuals of the model. It is well known that autocorrelation leads to statistical problems that may affect the quality of inference and policy prescriptions derived from the analysis [21,22]. We test for autocorrelation in the residuals of the model by means of Moran's *I *coefficient of spatial autocorrelation [23], implemented using a first order contiguity matrix **W **with element *w*_*ij *_= 1 if census tracts *i *and *j *share a length of border, and 0 otherwise. The matrix is row-standardized so that the sum of elements in a row is always exactly 1. The interpretation of a vector multiplied by a row-standardized matrix is as a moving average, since it gives for each location the average of the values in contiguous areas.

In order to address the issue of residual spatial autocorrelation, we propose to introduce a spatial filter to absorb any detected residual pattern [24]. The filtering approach used in this study is based on the eigenvectors of the following matrix:

(4)

where **I **is the identity matrix, **1 **is a *n *× 1 vector of ones, and **W **is the contiguity matrix of the system [25]. The eigenvectors represent patterns of latent autocorrelation, and combinations can usually be found that proxy omitted variables responsible for the residual pattern. The precise selection and number of eigenvectors for a filter depends on statistical criteria. Since eigenvectors are orthogonal, it is possible to follow a step-wise procedure whereby an eigenvector is introduced into the model and its significance tested. The first significant eigenvector (in our case the first one to attain a regression coefficient with a *p*-value of less than 0.10), is multiplied by its corresponding coefficient and introduced as an explanatory variable in the subsequent search for additional eigenvectors. Other significant eigenvectors are incorporated as part of the filter by summing them, after multiplying by their corresponding regression coefficient, to the previous version of the filter. The procedure continues until a desired level of spatial autocorrelation is reached (in our case, a non-significant z-score ≤ |0.05|). The spatial filter is a synthetic variable, not necessarily meaningful by itself, but useful to remove residual autocorrelation, which improves the statistical quality of the model, all the while helping to ensure that other coefficients in the model are not afflicted by omitted variable bias [26].

## Results

We estimate an initial model (Model 1) with a selection of variables from those shown in Table 2. The dependent variable is transformed using the natural logarithm operator. The dependent variables are not transformed. With few exceptions, the coefficients are significant at the *p *< 0.05 level, and have expected and/or reasonable signs. With respect to the demographic variables, the proportion of people in the 15-24 year range correlates positively with number of donors, as do the pre-retirement cohort of 55-64 (at a marginal level of significance of p = 0.0648). In contrast, as the proportions of working age population and seniors increase, the donor yield tends to decrease. Other variables that correlate positively with donor rates are the proportion of English speakers, highly educated individuals, and the proportion of people employed in health-related occupations. Intriguingly, the effect of wealth is negative: neighbourhoods with higher average household income tend to correlate with a lower number of donors. Similarly, although not unexpectedly, the proportion of immigrants correlates negatively with number of donors.

**Table 2:** Regression Model 1

**VARIABLE**	**ESTIMATE**	**p-value**	**vif**
CONST	-5.3594	0.0000	-
POP15TO24	2.1200	0.0000	1.19
POP25TO54	-0.3102	0.1060	0.83
POP55TO64	0.4833	0.0648	1.42
POP65+	-0.7670	0.0001	1.56
ENGLISH	1.3164	0.0000	3.67
IMMIG	-0.3025	0.0005	4.47
HIGHED	1.2928	0.0000	2.16
Ave Household Income ($10,000)	-0.0042	0.0424	1.73
Unemployment	-8.5297	0.0000	1.61
Health Occupations	2.2787	0.0004	1.64
Pop Based Access (4 km)	-0.0001	0.1871	1.48

CALGARY (AB)	0.4020	0.0000	1.31
EDMONTON (AB)	0.3484	0.0000	1.43
LETHBRIDGE (AB)	0.7668	0.0000	1.08
MEDICINAL HAT (AB)	0.2778	0.0030	1.05
RED DEER (AB)	0.5038	0.0000	1.07
KAMLOOPS (BC)	-0.3115	0.0001	1.07
KELOWNA (BC)	0.2961	0.0000	1.08
PRINCE GEORGE (BC)	0.5968	0.0000	1.08
VANCOUVER (BC)	0.0869	0.0001	1.29
WINNIPEG (MB)	0.5394	0.0000	1.33
FREDERICTON (NB)	0.6939	0.0000	1.07
MONCTON (NB)	0.7471	0.0000	1.17
SAINT JOHN (NB)	0.3886	0.0000	1.30
ST. JOHNS (NL)	0.5771	0.0000	1.26
HALIFAX (NS)	0.2439	0.0000	1.30
BARRIE (ON)	0.1815	0.0061	1.07
GUELPH (ON)	0.4340	0.0000	1.04
HAMILTON (ON)	0.1851	0.0000	1.23
KINGSTON (ON)	0.1578	0.0094	1.12
KITCHENER (ON)	0.4216	0.0000	1.13
LONDON (ON)	0.4961	0.0000	1.24
NORTHBAY (ON)	0.5870	0.0000	1.08
OSHAWA (ON)	0.1968	0.0000	1.14
OTTAWA (ON)	0.2935	0.0000	1.83
PETERBOROUGH (ON)	0.3333	0.0000	1.09
SARNIA (ON)	0.3200	0.0001	1.06
SAULT ST. MARIE (ON)	0.2152	0.0080	1.07
ST. CATHERINES (ON)	0.2572	0.0000	1.17
SUDBURY (ON)	1.1393	0.0000	1.20
THUNDER BAY (ON)	-2.9853	0.0000	1.14
WINDSOR (ON)	0.7685	0.0000	1.11
REGINA (SK)	0.6623	0.0000	1.15
SASKATOON (SK)	0.5078	0.0000	1.18

*n *= 3746, *R*^2 ^= 0.647, *σ*^2 ^= 0.153, *σ *= 0.394, *I *(*z*-score) = 0.2031 (22.0590)

Extensive analysis of different distance bands for the accessibility variables leads to the adoption of 4 km bands. This distance, although impossible to validate based on any empirical measure of travel behaviour, does not strike us as being unreasonable, since it represents a relatively short trip to the donation site from either the place of residence or the place of employment. Population-based accessibility, contrary to expectations, displays a negative sign, although the coefficient is not significant.

The fit of the model is fairly high, with the model explaining about 65% of the variance, although the large number of city-specific dummy variables (33 out of 39 candidates, not counting a reference city) indicates that there tend to be significant and substantial contextual effects. Spatial autocorrelation analysis of the residuals (which are in the same units as the dependent variable) indicates that unfortunately the assumption of independence cannot be sustained for this model. Calculation of variance inflation factors (vif) in contrast indicates that multicollinearity is not a problem.

In order to investigate the effect of metropolitan area effects and to address the issue of residual spatial pattern we estimate a second model. Autocorrelation is problematic because it can lead to bias and wrong inference. The second model is different from the first one in two important respects. First, we aim at capturing some of the contextual variation embedded in the city-specific dummy variables, and to this end we introduce CMA population as a macro-level descriptor of metropolitan areas. This variable is interacted with other explanatory variables in the model to produce expanded coefficients that consist of a direct and interaction effect that permits a mapping of the net effect as a function of the expansion variable. And secondly, we deal with residual pattern evident from autocorrelation analysis by means of the spatial filtering approach previously described. The results of the analysis appear in Table 3. Note that residual autocorrelation is effectively removed by the spatial filter. The goodness of fit has also improved somewhat, since now the model explains about 74% of the variance.

**Table 3:** Regression Model 2

**VARIABLE**	**ESTIMATE**	**p-value**	**vif**
CONST	-4.8351	0.0000	-
POP15TO24	1.5889	0.0000	1.12
POP55TO64	0.3920	0.0577	1.36
POP65+	-0.3936	0.0001	1.38
ENGLISH	0.7055	0.0000	2.03
IMMIG	-1.1326	0.0000	2.66
HIGHED	1.5693	0.0000	1.76
Ave Household Income ($10,000)	0.0127	0.0000	3.62
*CMA Population	-0.0049	0.0000	3.60
Unemployment	-5.1708	0.0000	1.40
Pop Based Access (4 km)	-0.0002	0.0827	2.45
*CMA Population	0.0005	0.0036	2.34

EDMONTON (AB)	0.1866	0.0000	1.16
LETHBRIDGE (AB)	0.4725	0.0000	1.04
REDDEER (AB)	0.4772	0.0000	1.04
KELOWNA (BC)	0.2515	0.0000	1.04
PRINCE GEORGE (BC)	0.3723	0.0000	1.04
WINNIPEG (MB)	0.1264	0.0000	1.10
FREDERICTON (NB)	0.2218	0.0011	1.04
MONCTON (NB)	0.8303	0.0000	1.08
HALIFAX (NS)	0.0742	0.0281	1.12
KITCHENER (ON)	0.7040	0.0000	1.06
LONDON (ON)	0.2216	0.0000	1.07
NORTHBAY (ON)	0.3275	0.0000	1.04
OSHAWA (ON)	0.6779	0.0000	1.09
OTTAWA (ON)	-0.1509	0.0001	1.42
PETERBOROUGH (ON)	0.0481	0.0563	1.03
SUDBURY (ON)	0.2668	0.0000	1.09
THUNDERBAY (ON)	-3.3342	0.0000	1.11
WINDSOR (ON)	0.3924	0.0000	1.07
REGINA (SK)	0.3950	0.0000	1.06

Filter	1.0000	0.0000	1.24

*n *= 3746, *R*^2 ^= 0.739, *σ*^2 ^= 0.113, *σ *= 0.338, *I *(*z*-score) = -0.0024 (0.4872)

The results clearly indicate that introducing the CMA population as a variable helps to express contextual variation in a more systematic way: the need for city-specific dummy variables is reduced from 33 to 19. The resulting model is more parsimonious and at the same time more informative. Interaction terms (linear and quadratic) with our CMA population variable were initially attempted for all explanatory variables; all non-significant coefficients were dropped from the analysis. The results for non-expanded coefficients are in line with the previous model, positive for proportion of people aged 15-24 and 55-64 years (the latter with a marginally significant value of p = 0.0577), negative for proportions of seniors, unemployed, and immigrants, and positive for proportion of English speakers and highly educated population. Two variables further lose their significance in this analysis: proportion of people 25-54 years and those in health-related occupations. In addition to reducing the number of dummy variables, using CMA population to estimate expanded coefficients also provides some valuable insights into the contextualizing effect of city size on two correlates of blood donation: Average Household Income and Population-based Accessibility. The first variable with a significant expanded coefficient is that corresponding to average household income. This variable was negative in Model 1. Now, the direct effect is seen to be positive and significant, which is more in line with our prior expectations regarding the effect of wealth. Furthermore, the net effect at a given level of the CMA population variable can be calculated as follows:

(5)

**Figure 3:**
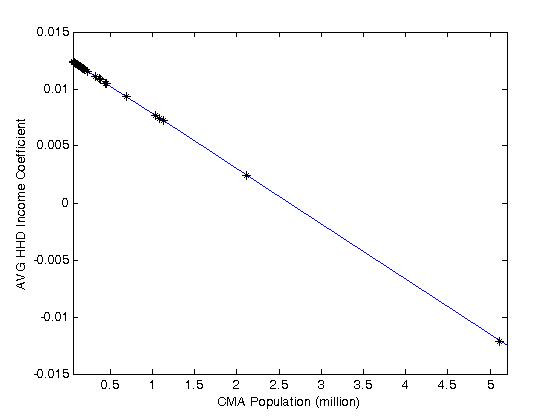
**Average Household Income coefficient as a function of CMA population**.

The net effect, shown in Figure 3, reveals an interesting pattern of variation according to city size. Whereas a positive and relatively large association is observed for census tracts in smaller cities, the effect tends to vanish with increasing city size, in fact becoming negative for the largest cities in the system. Thus, whereas wealthy census tracts in smaller cities tend to be more generous in their yield of donors, this is less, or not at all, the case in bigger cities. This is what could be termed a "stingy big-city" effect.

The accessibility variable based on population, which was negative but not significant in Model 1, is found to display significant city size variations, with a non-linear net effect that is calculated as follows:

(6)

**Figure 4:**
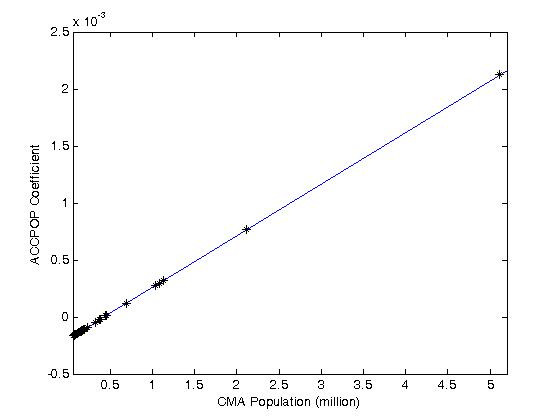
**Population-based (4 km) Accessibility coefficient as a function of CMA population**.

The (negative) direct effect in this case is revealed to be non-significant, and there is evidence that the effect of accessibility increases with size of population: as seen in Figure 4, improvements in access to levels of service are likely to have a greater impact the bigger the city is.

## Discussion

The analysis indicates that there are a number of common factors that influence donor turnout rates across Canadian metropolitan areas. Some of the factors, furthermore, are shown to display systematic variations according to one contextualizing factor, namely CMA population. The results highlight some of the challenges and opportunities facing the blood supply system in Canada. First, the demographics present a significant challenge, with the most generous age group being the 15-24 year cohort. This is a group that, due to long-term demographic trends will shrink both in absolute and relative terms, at the same time that the senior population (which correlates negatively with donor turnout rates) tends to increase. With respect to the younger population, it is unclear if cultural factors are in play to make a more active cohort remain so over their lifetime, and it is possible that newer younger generations will continue to produce more donors than the current working-age population (25-54 years). On the contrary, if limitations to donor behaviour are due not primarily due to cultural factors but rather to other circumstances, for example more strict time constraints as this population integrates more fully as part of the workforce, then a different challenge arises in terms of finding ways to continue to support the altruistic behaviour of young donors as they enter the workforce, for example by increasing the convenience and levels of service at employment-rich locations.

A second result that seems to suggest a challenge is the negative relationship between proportion of immigrants and donor rates, given the importance of immigration in Canadian demographic processes. It could be argued that the effect of immigrants on number of donors is coloured by variations in income, education levels, or language proficiency (q.v. the positive effect of English). A cautious argument, mindful of the potential for committing an ecological fallacy, is that since these variables have already been controlled for in the model, the negative relationship could rather be attributed to cultural factors and attitudes. It is important to note that immigrants are not by any means a homogenous group, and in fact there could be important differences between various immigrant populations. Clearly, more research would be needed to determine the extent to which immigrant's donor behaviour is different from the host population due to socio-economic and cultural reasons.

Education appears to be an important factor that influences donor yield. The positive coefficient for this variable is second only to that associated with the proportion of unemployed population in terms of magnitude. This seems to confirm the importance of education in motivating donors, and it is possible that the objectives of marketing campaigns are easier to comprehend by highly educated individuals. A suggestion would be to target marketing efforts to this population, or to device marketing strategies that more directly speak to segments of the population with various educational attainment levels. Household income tends to have a smaller positive impact or to become negative for larger population centres.

Population-based accessibility to donor clinic services stands out as an important policy variable that needs to be considered, in particular in light of contextual effects that indicate a positive relationship between city size and accessibility.

Our attempt to contextualize the effect of various correlates of donor turnout rates resulted in a more parsimonious and informative model that produced a better fit while resorting to a smaller number of city-specific dummy variables. Out of 39 possible dummy variables, the first model used 33 dummy variables which included all of the Prairie and Atlantic CMAs. Thus, the initial model, while appearing to have significant strength in explaining the variation of donor behaviour across Canada, failed to capture more in full the donor patterns for these regions. Within Ontario, the cities represented are the North-Eastern CMAs such as Sudbury and North Bay as well as some of the cities that contain parts of the main 400 series and QEW highways around Lake Ontario, although beyond these observations there appears to be no definitive pattern within Ontario. In British Columbia the initial model includes only the suburban cities as the dummy variables represent Vancouver and the more rural CMAs in the Eastern parts of the province.

The second model in contrast reduced the need for dummy variables (19) and accounted for two Atlantic CMAs as well as seven Prairie cities. The model also better represents British Columbia by having dummy variables only for some of the Northern CMAs. While the number of Ontario cities included in the model decreases from 16 to 9, the regional representation in the second model is not much different from the initial model where there is no clear pattern in the distribution of dummy variable cities across the province.

In this paper our objective has been to develop a big picture of the factors influencing donor turnover rates, and the analysis has successfully identified common factors and, where warranted, their variability according to city size. The continued existence of significant contextual effects, on the other hand, suggests that further analysis at the metropolitan level is necessary.

## Conclusion

Blood products are an essential component of modern medicine and necessary to support many life-saving and life-prolonging procedures. CBS has successfully managed to build an active donor base of approximately 425,000 donors and 916,000 whole blood donations in 2008. However, the total number of donors and donations still needs to be increased in order to meet longer term projections of demand [3]. Considering the fact that turnout rates are but a small proportion of the potential donor pool, concerted action is called for in order to ensure the sustainability of a system that currently relies on a small number of Canadians to provide for the whole country. Actions will necessarily involve campaigns designed to encourage a greater the number of Canadians to adopt blood donation as part of their lives, and to facilitate the practice.

The ideal answer to the dilemma of how to increase the nation's supply of blood is to increase the number of new donors and to retain them as repeat donors. This requires an understanding of the correlates of blood donation across the country. In order to support new efforts to encourage blood donation, in this paper we investigated the correlates of donation to determine the factors that may influence donor turnout rates. The results indicate that a number of significant correlates of blood donor turnout rates behave consistently across geographical regions and urban sizes. In some cases, in contrast, patterns of variation across cities of various sizes were detected. Understanding these patterns can assist in the economical efficiency of any marketing plan as well as maximize results by ensuring that both message and services target individuals in each city who are most likely to donate blood.

A number of suggestions and directions for further research are indicated as follows. As previously indicated, it is possible to incorporate travel behaviour information directly into the assessment of catchment areas. Lacking this, in our analysis we have opted for a purely statistical strategy for selecting accessibility bands, and while the results are reasonable, they may or may not reflect the actual distance that people who donate are willing to travel to reach a donor clinic. An important constraint is that at the moment donor's information is collected that asks for place of residence but not place of employment, nor whether the trip to donate was home- or non-home-based. It is therefore currently not possible to determine, for instance, the typical trip length of a donor visiting a clinic. Relatively simple changes to the way data is collected should help to develop a better understanding of the conditions surrounding trips to donor clinics. Along this way lay more refined estimates of catchment areas that could in fact be different for home-based (i.e. residential population) and non-home-based (i.e. employment-based accessibility). This information is relevant for obvious operational reasons.

Along with the spatial characteristics of donor clinics (i.e. their location), another factor of interest is the possibility that younger cohorts are more likely to donate due to cultural or other factors. If time constraints are in fact responsible for the reduced participation of the working-age population, it would be important to understand the time use patterns of people in this age cohort, in order to fine tune, for instance, the hours of operation of donor clinics, in addition to their locations. Presently, to the best of our knowledge, there is no research available on the time use patterns of donors, and CBS does not collect time use information. Collection of time use data appears a promising way to better understand the context of donation. The analysis presented in this paper, being at the aggregate level, does not lend itself to the study of time use patterns, however, and other methods useful to investigate behaviour at the level of the individual would be indicated. Individual level analysis would have in addition, the benefit of circumventing the ecological fallacy, and would provide better tools to disentangle the effect, for instance, of immigration status, income, English proficiency, education, etc. [27] This is a matter of ongoing research.

## Competing interests

The authors declare that they have no competing interests.

## Authors' contributions

AP, KBN, and NMH conceived of the study. AP secured access to the data. PJS performed the statistical analysis under the supervision of AP and KBN. PJS produced a first draft of the manuscript that was finalized by AP, KBN, and NMH. All authors read and approved the final manuscript.
